# Supplementary material for: Bioorthogonal Labeling Enables In Situ Fluorescence Imaging of Expressed Gas Vesicle Nanostructures
Source: Bioconjug Chem. 2024 Feb 12;35(3):333–9. doi: 10.1021/acs.bioconjchem.3c00518 (PMC10961726; doi:10.1021/acs.bioconjchem.3c00518)
Supplement: Supplementary file 1 — bc3c00518_si_001.pdf [file bc3c00518_si_001.pdf]

Supporting Information

Bioorthogonal Labeling Enables In Situ Fluorescence Imaging of Expressed Gas Vesicle Nanostructures

Erik Schrunk[1], Przemysław Dutka[1,2,†], Robert C. Hurt[2], Di Wu[1,*], and Mikhail G. Shapiro[1,3,4,*]

[1]Division of Chemistry and Chemical Engineering, California Institute of Technology; Pasadena, California 91125, United States
[2]Division of Biology and Biological Engineering, California Institute of Technology; Pasadena, California 91125, United States
[3]Andrew and Peggy Cherng Department of Medical Engineering, California Institute of Technology; Pasadena, California 91125, United States
[4]Howard Hughes Medical Institute; Pasadena, California 91125, United States
[†]Present address: Department of Structural Biology, Genentech Inc., South San Francisco, California 94080, United States
*Email: Di.Wu@caltech.edu. Phone: (626)395-8560.
*Email: mikhail@caltech.edu. Phone: (626)395-8588.

Contents:

Supplementary Figures S1-S4 and Supplementary Methods 1.

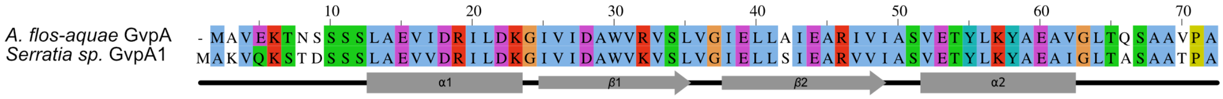

Figure S1: Sequence alignment for *Anabaena flos-aquae* gvpA and *Serratia sp. 39006* gvpA1. The two genes have an identity score of 80.6% (58 of 72 amino acids) and a similarity score of 91.7% (66 of 72 amino acids). Colored amino acids represent pairs of amino acids with similar or identical side chains: blue for hydrophobic groups, purple for negatively charged groups, green for polar uncharged groups, red for positively charged groups, orange for glycine, teal for aromatic groups (tyrosine and histidine), and yellow for proline.

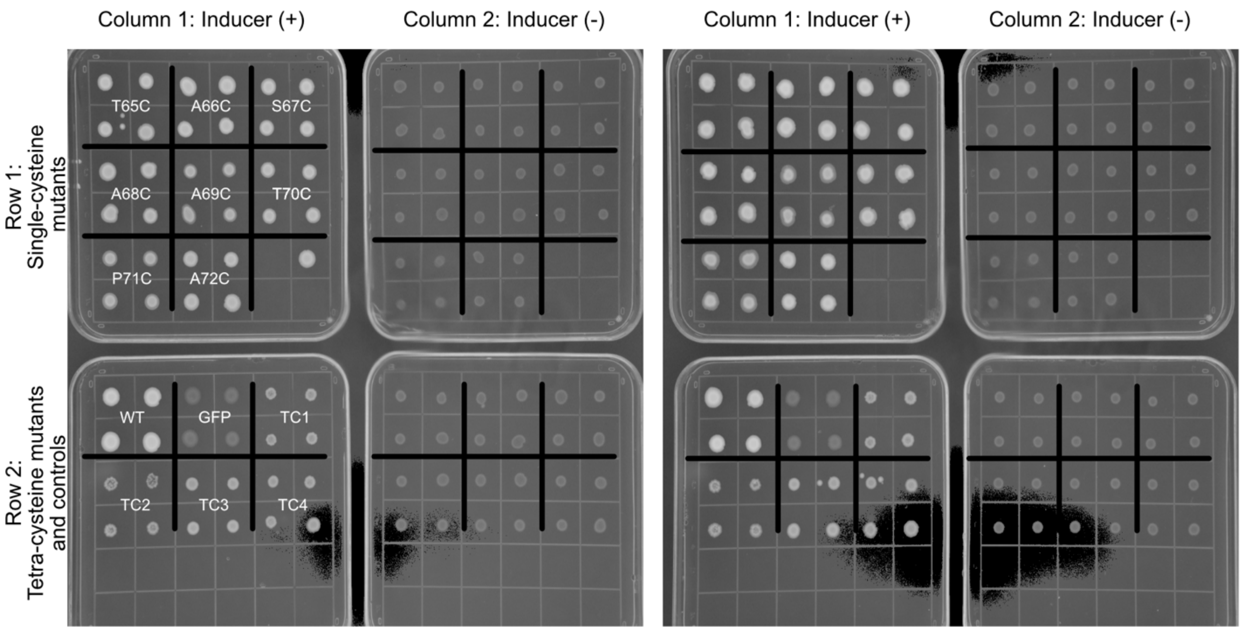

Figure S2: Opacity screen of mutant gvpA1 plasmids in *E. coli*. Patches of *E. coli* transformed with arabinose-inducible constructs encoding mutant gvpA1, wild type gvpA1, or GFP were grown on LB agar plates. Two images, each showing a set of four distinct plates, are shown. The patches on the four plates on the right represent duplicate technical replicates of the corresponding patches on the four plates on the left. Within each set of four plates, the two plates on the left (column 1) contain the inducer arabinose; the two plates on the right (column 2) do not. Each gvpA1 variant (and GFP) is represented by 4 patches arranged into a 2x2 grid in which each of the 4 patches originates from a different colony from the original transformation (*i.e.*, each of the 4 patches represents a distinct biological replicate). 2x2 grids of patches are labeled with their corresponding gvpA1 variant (or GFP) in the column on the left; all other columns are arranged identically.

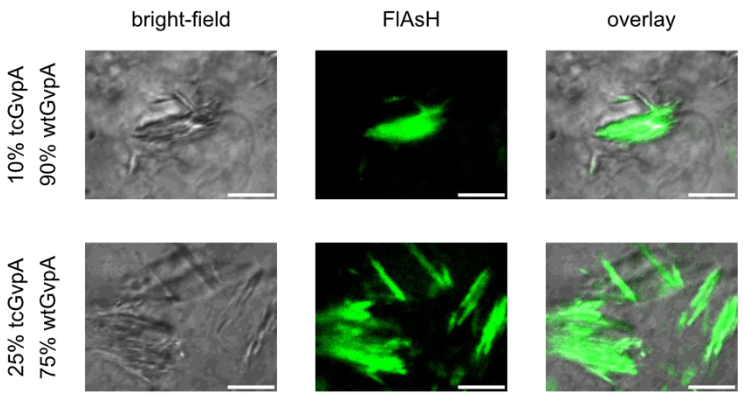

Figure S3: Images of FlAsH-labeled GVs in cells transfected with different ratios of wtGvpA:tcGvpA. Images of tcGV clusters in fixed HEK 293T cells transfected with 10% (top row) and 25% (bottom row) tcGvpA. GV clusters are visible under bright-field imaging (first column) and are brightly labeled with FlAsH (second column). The bright-field/FlAsH overlay (third column) demonstrates that the strongest FlAsH signal overlaps with tcGV clusters. All scale bars 5 μm. Cells transfected with 100% tcGvpA did not result in visible GV formation (data not shown).

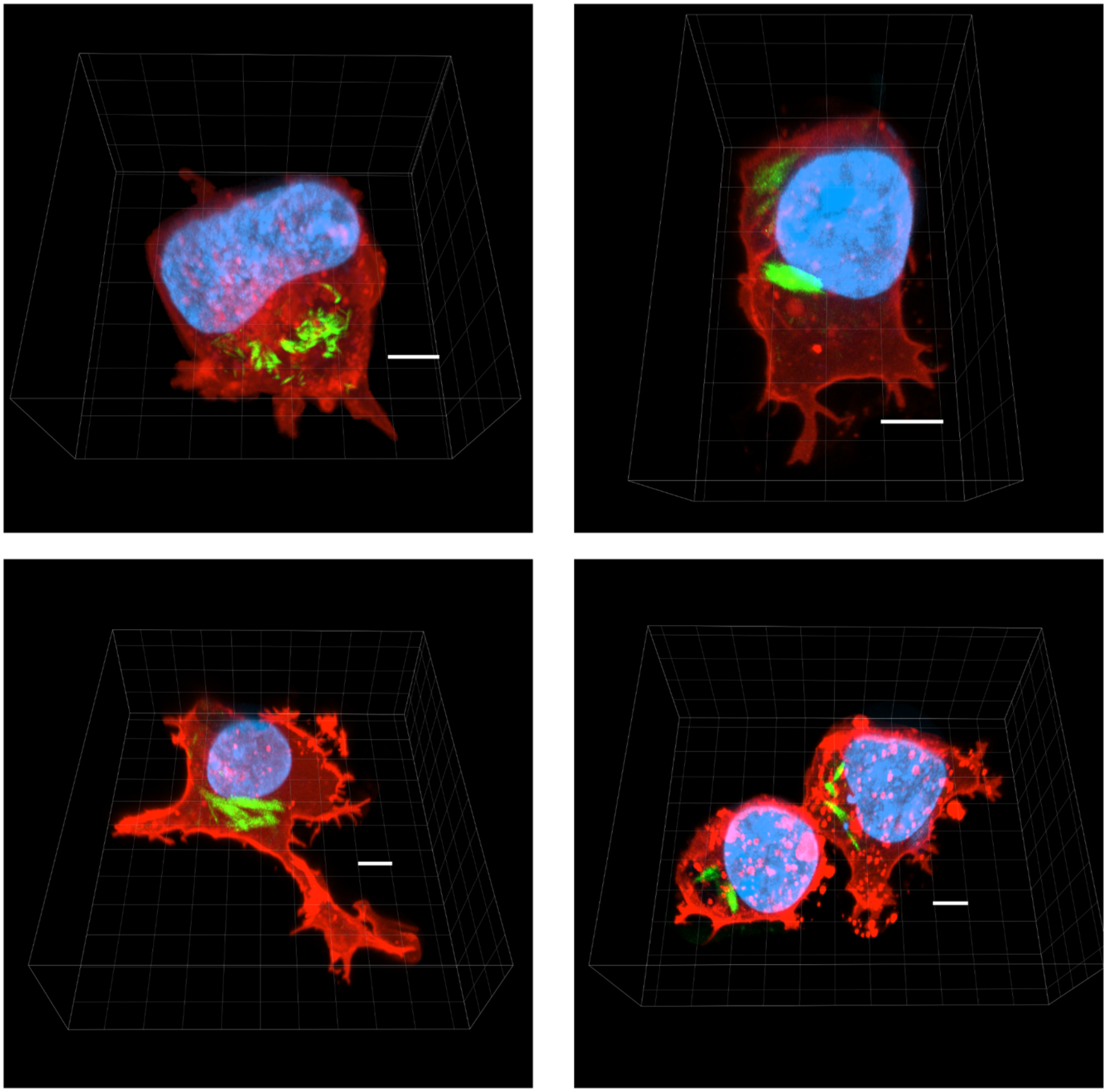

Figure S4: 3D renderings of tcGV-expressing cells. The cell membrane is depicted in red (Lck-mScarlet-I), tcGVs in green (FlAsH), and nucleus in blue (DAPI). All scale bars 5 µm.

Supplementary Methods 1: Estimation of the GvpA concentration within a typical FlAsH imaging voxel.

To estimate the molar concentration of GvpA in a typical imaging voxel, we first estimated the number of GvpA molecules in a typical GV (n_{GvpA}) using the length of a typical *Anabaena flos-aquae* GV ($L = 500nm$) (ref. 1), the number of GvpA per helical turn ($n = 227$) (ref. 2), and the helical pitch ($P = 4.9nm$) (ref. 2) using:

$$n_{GvpA} = n * L/P \approx 2 \times 10^4$$

Assuming a typical imaging voxel of $(500nm)^3$ for FlAsH imaging, a single GV within the voxel would result in a GvpA concentration of $\sim 300\ \mu M$. A tightly packed GV cluster within the voxel would give a GvpA concentration of $\sim 10\ mM$.

(1) Dutka, P., Metskas, L. A., Hurt, R. C., Salahshoor, H., Wang, T.-Y., Malounda, D., Lu, G. J., Chou, T.-F., Shapiro, M. G., Jensen, G. J. (2023) Structure of Anabaena Flos-Aquae Gas Vesicles Revealed by Cryo-ET. Structure, 31 (5), 518-528.e6.
(2) Huber, S. T., Terwiel, D., Evers, W. H., Maresca, D., Jakobi, A. J. (2023) Cryo-EM Structure of Gas Vesicles for Buoyancy-Controlled Motility. Cell, 186 (5), 975-986.e13.
